# Folic Acid and Methyltetrahydrofolate Supplementation in the *Mthfr^677C>T^* Mouse Model with Hepatic Steatosis

**DOI:** 10.3390/nu17010082

**Published:** 2024-12-28

**Authors:** Karen E. Christensen, Marie-Lou Faquette, Daniel Leclerc, Vafa Keser, Yan Luan, Jeanna L. Bennett-Firmin, Olga V. Malysheva, Alaina M. Reagan, Gareth R. Howell, Marie A. Caudill, Teodoro Bottiglieri, Rima Rozen

**Affiliations:** 1 Departments of Human Genetics and Pediatrics, McGill University, Montreal, QC H3A 0C7, Canada; 2 The Research Institute of the McGill University Health Centre, Montreal, QC H4A 3J1, Canada; 3 Center of Metabolomics, Institute of Metabolic Disease, Baylor Scott and White Research Institute, Dallas, TX 75204, USA; 4 Division of Nutritional Sciences, Cornell University, Ithaca, NY 14853, USA; 5 The Jackson Laboratory, Bar Harbor, ME 04609, USA

**Keywords:** folic acid, methyltetrahydrofolate, supplementation, MTHFR, hepatic steatosis, mouse model

## Abstract

Background/Objectives: The *MTHFR*^677C>T^ gene variant results in a thermolabile MTHFR enzyme associated with elevated plasma homocysteine in TT individuals. Health risks associated with the TT genotype may be modified by dietary and supplemental folate intake. Supplementation with methyltetrahydrofolate (methylTHF) may be preferable to folic acid because it is the MTHFR product, and does not require reduction by DHFR to enter one-carbon folate metabolism. In the *Mthfr^677C>T^* mouse model for this variant, female 677TT (TT) mice have an increased incidence of hepatic steatosis. The objective of this study was to compare the effects of methylTHF and folic acid supplementation on hepatic steatosis and one-carbon metabolism in this model. Methods: Male and female C57BL/6J 677CC (CC) and TT mice were fed control (CD), 5xmethylTHF-supplemented (MFSD), or 5xfolic-acid-supplemented (FASD) diets for 4 months. Liver sections were assessed for steatosis by Oil Red O staining. One-carbon metabolites were measured in the liver and plasma. MTHFR protein expression was evaluated in the liver. Results: MFSD had no significant effect on plasma homocysteine, liver SAM/SAH ratios, or hepatic steatosis in males or females as compared to CD. MTHFR protein increased in MFSD TT female liver, but remained <50% of the CC. FASD had no effect on plasma homocysteine but it decreased the liver MTHFR protein and SAM/SAH ratios, and increased hepatic steatosis in CC females. Conclusions: MethylTHF and folic acid supplementation had limited benefits for TT mice, while folic acid supplementation had negative effects on CC females. Further investigation is required to determine if these effects are relevant in humans.

## 1. Introduction

Methylenetetrahydrofolate reductase (MTHFR), a key enzyme in one-carbon metabolism, commits folate to the methylation cycle by reducing 5,10-methylenetetrahydrofolate to 5-methyltetrahydrofolate (methylTHF) (see pathway: Figure S1) [1]. MethylTHF production by MTHFR supports the detoxification of homocysteine (Hcy) by methionine synthase (MTR) through its remethylation to methionine. The *MTHFR*^677C>T^ gene polymorphism is a common variant of MTHFR which has been associated with the increased risk for several disorders, including birth defects and stroke [2,3,4,5]. This gene variant encodes an alanine-to-valine substitution at position 222 (A222V) that impairs enzyme function and leads to elevated homocysteine in 677TT individuals [6,7].

The A222V substitution results in a form of MTHFR that is more susceptible to loss of activity when heated. This thermolabile form of MTHFR can be stabilized in vitro by the addition of its methylTHF product or its FAD cofactor [8]. Consistent with these observations, the increased Hcy concentrations in 677TT individuals are greater in those with low folate than those with high folate levels [9,10], and disease risks associated with 677TT have been reported to be modified by folate intake [11,12]. For this reason, folate supplements are often recommended for 677TT individuals.

Folic acid is the form of folate most commonly used in supplements [13]. In recent years, methylTHF supplements have become widely available, which has led to increased discussion as to which folate form is preferable. MethylTHF may be advantageous as a supplement because it does not require reduction by dihydrofolate reductase (DHFR) to be active, unlike folic acid [14]. It has been suggested that methylTHF supplements may be more beneficial than folic acid to the health of 677TT individuals because it circumvents the loss of MTHFR activity, but there has been little research into this area to date [15].

In studies comparing these supplements, folic acid and methylTHF have been equally effective at lowering plasma Hcy, and methylTHF has been equivalent or better at raising plasma and red blood cell folates [14,16,17]. It is not clear how the effects of these supplements may differ in organs such as the liver, because plasma measurements do not necessarily correspond to levels in tissues. The liver is the major site of folate metabolism in the body [18]; therefore, one goal of this study was to compare the effects of folic acid and methylTHF supplementation on metabolites in this tissue.

One major difference between the two supplements is the presence of unmetabolized folic acid (UMFA) in the circulation due to supplemental folic acid [14]. MethylTHF is the primary form of folate in the circulation; other forms are generally only observed when folate metabolism is disrupted. Folic acid requires a reduction by DHFR to be converted to biologically active tetrahydrofolate (THF). DHFR may be saturated by excess folic acid from supplements, leading to the appearance of UMFA in plasma [19,20]. There are suggestions that excess UMFA may have adverse effects on human health; a variety of adverse outcomes have been reported in rodents fed high-folic-acid diets [21,22,23,24,25,26,27,28]. More research is required to evaluate these effects in humans, and to distinguish the effects of UMFA, in particular, from those of high total folate intake [29].

The MTHFR heterozygous knockout mouse (*Mthfr^+/−^)* has been used as a model for the reduced MTHFR activity in 677TT individuals, but, as it does not have the thermolabile form of MTHFR, this model cannot reproduce the potential stabilizing interactions with nutrient intake [30]. The new *Mthfr^677C>T^* mouse model was created using CRISPR to insert the alanine-to-valine mutation that is equivalent to the human A222V variant [31]. *Mthfr*^677CC^ (CC) and *Mthfr*^677TT^ (TT) mice model humans with the *MTHFR*^677CC^ and *MTHFR*^677TT^ genotypes, respectively, with respect to the reduced MTHFR activity, enzyme thermolability, and increased plasma homocysteine. In our recent work, we found that female TT mice had increased susceptibility to hepatic steatosis that was aggravated by folate deficiency [30]. Hepatic steatosis, the accumulation of lipid droplets in liver, is part of the spectrum of metabolic-dysfunction-associated steatotic liver disease (MASLD, formerly NAFLD). Female TT mice had an altered expression of lipid metabolism genes that could impair VLDL synthesis or export and result in steatosis [30]. In a meta-analysis of human studies, the MASLD risk was positively associated with Hcy and negatively associated with serum folate, leading to the suggestion that adequate folate intake may prevent MASLD [32].

In this study, we compared the effects of supplementation with folic acid and methylTHF in this new model, which permits the direct examination of the effects of these supplements in the liver, an organ that is not accessible in human studies. We compared a control diet containing the recommended amount of folic acid for mice with diets supplemented with 5× the recommended amount of folic acid or the equivalent amount of methylTHF. Our objectives were to determine whether methylTHF had any specific metabolic benefits in liver, as compared to folic acid, and whether folate supplements would reduce the steatosis risk in TT females.

## 2. Materials and Methods

### 2.1. Mice and Diets

Animal experimentation was performed following Canada Council on Animal Care guidelines and approved by the RI-MUHC Animal Care Committee (AUP 3132). *Mthfr*^677C>T^ C57BL/6J mice were bred in-house and genotyped as in [30]. Mice were group-housed with ad libitum access to food and water in randomly distributed cages in the same room, with a 12:12-h light-dark cycle, at 18–24 °C under specific pathogen-free conditions. Four-week-old male and female CC and TT mice were randomly assigned to control (CD, 2 mg folic acid/kg diet, as recommended for rodents), folic-acid-supplemented (FASD, 10 mg folic acid/kg diet), or methylTHF-supplemented diets (MFSD, 0.3 mg folic acid + 10.1 mg calcium L-methylTHF/kg diet), with 14–30/group to obtain sufficient liver samples for all experiments. FASD and MFSD have equimolar folate contents and have the same base formulation as CD. These amino-acid-defined diets were produced by Inotiv (Madison, WI, USA), and the formulations are shown in Table S1.

FASD with 5 times the recommended amount of folic acid has been used in previous studies to approximate high folate intakes observed in human populations [21,24]. MFSD has the same folate concentration in moles as FASD to allow the direct comparison of the effects of the two supplements. MFSD contains both folic acid and methylTHF to approximate human folate intake, which would include small amounts of folic acid from fortified foods as well as the methylTHF supplement. There were no significant differences between the consumption of the 3 diets. On average, male mice consumed 2.6 g/day of CD, 2.7 g/day of MFSD, and 2.7 g/day of FASD, and females consumed 2.3 g/day of CD, 2.4 g/day of MFSD, and 2.3 g/day of FASD. The diets and/or genotypes of the mice did not show significant differences in body weights, liver weights (as % body weight), or any obvious signs of ill health.

After 4 months on diets, the mice were euthanized by CO_2_ asphyxiation under isoflurane anesthesia. Blood was collected by cardiac puncture in lithium-heparin collection tubes. Plasma was harvested by centrifugation and snap-frozen on dry ice. The liver was dissected, rinsed in cold phosphate-buffered saline, and weighed; the left lobe was fixed in 4% paraformaldehyde and the remaining liver was snap-frozen. Frozen plasma and liver were stored at −80°C, and were thawed only one time for analysis.

### 2.2. MTHFR Activity Assays

Preparation of crude liver extracts from male mice fed standard lab chow, and ^14^C-methylTHF-menadione oxidoreductase MTHFR assays were performed as in [31]. The extraction buffer contained 50 mM potassium phosphate pH 7.2, 50 mM sucrose, 0.3 mM EDTA, Pierce protease inhibitors and phosphatase inhibitors (ThermoFisher, Waltham, MA, USA) to preserve enzyme phosphorylation. To evaluate stabilization by FAD or folates, protein samples were equilibrated to 37 °C for 5 min, then incubated at either 37 or 46 °C for 10 min with or without the stabilizing agent (either 35 µM FAD, 100 µM methylTHF, or 100 µM THF) before assaying MTHFR activity. Reactions were performed in duplicate for each temperature/concentration condition with 1 blank, using crude liver extracts from 3–4 CC and TT mice each. For the methylTHF assays, the concentration of ^14^C-methylTHF in the assay was increased from 200 to 300 µM to offset dilution of the labeled substrate by cold methylTHF.

### 2.3. Metabolite Measurement in Plasma and Liver

5-MethylTHF, folic acid, and total homocysteine were measured in plasma by LC-MS as described in [33,34]. Liver SAM and SAH were quantified by stable isotope-dilution LC-electrospray ionization tandem MS as described [30,35]. Hepatic methionine, choline, betaine, and dimethylglycine were determined by LC MS/MS as in [30,36].

### 2.4. Histological Assessment of Hepatic Steatosis

Fixed liver was frozen in OCT and 10 μm sections were stained with Oil Red O (RI-MUHC-Histopathology Platform). Two sections per mouse were scored for the presence of Oil-Red-O-stained lipid droplets [37] at 10× magnification by two individuals blinded to genotype, diet, and sex. Steatosis severity was graded based on the percentage of the total tissue area affected with visible lipid droplets and categorized as <5%, 5–33%, 33–66%, or >66%, as in [37].

### 2.5. Western Blotting

Protein extraction and immunoblotting were performed as in [21] using primary antibodies specific for MTHFR [6] and ACTIN (A2066; Sigma-Aldrich, Oakville, ON, Canada). The RIPA extraction buffer contained Pierce protease inhibitors and phosphatase inhibitors (ThermoFisher). Immunoreactive protein was visualized with Radiance Q chemiluminescent substrate (Azure Biosystems, Dublin, CA, USA). Blots were imaged and quantified using the Amersham Imager 600 (GE Healthcare Life Sciences, Chicago, IL, USA, analysis software v1.0.0). MTHFR was normalized to the ACTIN loading control for each sample. Normalization by ACTIN was validated by comparing to normalization by amido black protein staining on the membrane (*p* < 0.0001, *r* = 0.74). As multiple blots were required, samples were divided equally between the blots and the data were combined by normalizing to the CD CC group on each blot.

### 2.6. Quantitative RT-PCR (qRT-PCR)

Total RNA was isolated from frozen liver using the Aurum Total RNA mini kit and reverse transcribed with the iScript cDNA Synthesis kit (both Bio-Rad, Hercules, CA, USA). Quantitative real-time PCR was conducted using SsoAdvanced Universal SYBR Green Supermix (Bio-Rad) on a LightCycler 480 II (Roche Diagnostics, Basel, Switzerland). Normalization factors were calculated using *Sdha* and *Psmc4* as reference genes with geNorm v.3.4 [38]. Primers without citations were designed using Primer-BLAST (https://www.ncbi.nlm.nih.gov/tools/primer-blast/, accessed on 24 December 2024) [39]. Genes, primer sequences, and reaction conditions are shown in Table S2.

### 2.7. Statistical Analysis

Continuous data were analyzed using 2-way ANOVA followed by Tukey post hoc analysis, or by 1-way ANOVA with post hoc analysis for trend. Significant statistical outliers (no more than one per subgroup) were identified by using Grubbs’ test and removed from the analysis. Histology scores were analyzed by ordinal regression (MASS package [40]) using R 4.3.1 [41] and R Studio 2024.04.2 [42] software. Meaningful genotype–diet interactions to include in regression models were determined by lowest Akaike information criterion (AIC). Individual mice were used as the experimental unit for calculations. Male and female data were analyzed separately. Adequate sample sizes were determined based on previous experiments with these mice to estimate expected effect sizes and variances [30,31]. Unless otherwise noted, analyses were performed using Graphpad Prism 8.0.1. Values are presented as mean ± SEM. For all analyses, *p* ≤ 0.05 was considered significant; *p* ≤ 0.07 was considered a trend.

## 3. Results

### 3.1. Stabilization of the Mouse Variant 677TT MTHFR by FAD and Folates Is Consistent with Human

**Figure 1:**
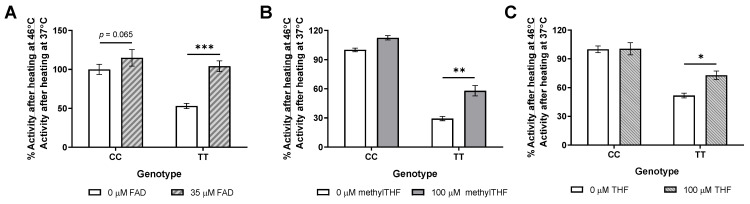
*Mthfr*^677C>T^ mouse TT protein is stabilized by FAD and folates. Activity after heating, expressed as percent activity of CC samples at 0 μM, after pre-incubation with (**A**) FAD (*p_FAD_* = 0.043, *p_TT_* = 0.0004, *p_interaction_* = 0.0055), (**B**) methylTHF (*p_methylTHF_* = 0.0017, *p_TT_* < 0.0001, *p_interaction_* = 0.079), and (**C**) THF (*p_THF_* = 0.038, *p_TT_* = 0.0002, *p_interaction_* = 0.045). 3-4 biological replicates per group, assayed in duplicate; 2-way repeated-measures ANOVA, Šidák post hoc * *p* < 0.05, ** *p* < 0.005, *** *p* < 0.001.

The MTHFR A222V variant protein is a thermolabile enzyme that is associated with reduced MTHFR activity after heating, in individuals homozygous for the 677C>T SNP [6]. The human 222VV protein has been shown to be stabilized during heating by methylTHF and FAD in human lymphocyte extracts and in purified recombinant protein [8]. The stabilization of the mutant mouse protein by FAD, methylTHF, and THF was assessed in liver extracts from chow-fed male mice (Figure 1). The MTHFR TT activity was restored to near wild-type levels by 35 µM FAD (Figure 1A), and was almost doubled by 100 µM methylTHF (Figure 1B). The MTHFR activity also increased by 41% due to the 100 µM THF (Figure 1C), suggesting that different forms of supplemental folate could help to stabilize the TT protein.

### 3.2. MFSD and FASD Increase Plasma Folates, but Do Not Decrease Total Homocysteine

**Figure 2:**
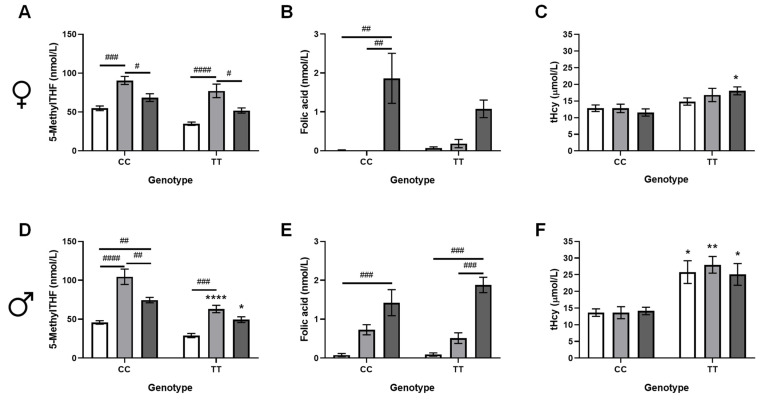
Effects of diet and genotype on plasma folates and total homocysteine (tHcy). In females: (**A**) methylTHF (*p_TT_* = 0.0004, *p_diet_* < 0.0001, *p_interaction_* = 0.82), (**B**) unmetabolized folic acid (*p_TT_* = 0.46, *p_diet_* < 0.0001, *p_interaction_* = 0.23), (**C**) tHcy (*p_TT_* = 0.0006, *p_diet_* = 0.71, *p_interaction_* = 0.26). In males: (**D**) 5-methyltetrahydrofolate (methylTHF; *p_TT_* < 0.0001, *p_diet_* < 0.0001, *p_interaction_* = 0.067), (**E**) unmetabolized folic acid (*p_TT_* = 0.57, *p_diet_* < 0.0001, *p_interaction_* = 0.18), and (**F**) total homocysteine (tHcy; *p_TT_* < 0.0001, *p_diet_* = 0.86, *p_interaction_* = 0.77). n = 5–6/group; 2-way ANOVA, Tukey post hoc: * CC vs. TT: * *p* < 0.05, ** *p* < 0.005, **** *p* < 0.0001; # diet comparison: ^#^ *p* < 0.05, ^##^ *p* < 0.005, ^###^ *p* < 0.001, ^####^ *p* < 0.0001. Control diet (CD): white bars; methylfolate-supplemented diet (MFSD): light grey bars; folic-acid-supplemented diet (FASD): dark grey bars.

MethylTHF and folic acid in plasma were measured to compare the effects of the equimolar supplemental diets on circulating folates (Figure 2). In individual comparisons, circulating methylTHF was lower in CD-fed TT mice of both sexes as compared to CD CC mice, as expected (females *p* = 0.0003, males *p* = 0.0007, *t*-test). In MFSD-fed mice, plasma methylTHF roughly doubled in mice of both genotypes and sexes (Figure 2A,D). Plasma methylTHF also increased in FASD-fed mice, but to a lesser extent than with MFSD. In spite of these increases, plasma methylTHF remained significantly lower in TT mice of both sexes, regardless of folate intake (Figure 2A,D).

Unmetabolized folic acid was significantly increased in the plasma of FASD-fed mice of both sexes, and was not affected by the MTHFR genotype (Figure 2B,E). Folic acid was also detected in the plasma of CD and MFSD males of both genotypes. Although it is interesting that more folic acid was found in MFSD mice than CD mice, considering that MFSD contains less folic acid than CD, it should be noted that the amount of folic acid detected in CD and MFSD mice is below the limit of detection of 1 nmol/L, and, therefore, cannot be reliably determined.

Plasma homocysteine was significantly higher in TT males and females (Figure 2C,F), as expected; however, there were no differences in tHcy between dietary groups. This finding suggests that folate supplementation over the amount in CD may have no effect on homocysteine metabolism.

### 3.3. Hepatic Steatosis in Female Mice Is Affected by 677TT Genotype and Nature of Folate Supplement

**Figure 3:**
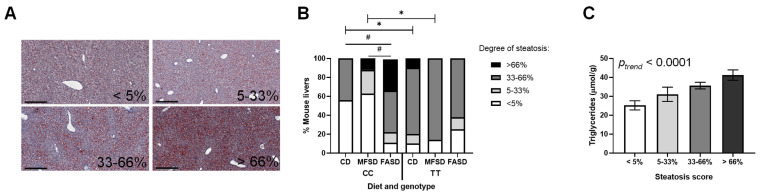
Steatosis in female mice is affected by folate intake and 677TT genotype. (**A**) Degree of steatosis was scored as % area affected of Oil-Red-O-fixed-frozen sections by 2 blinded observers. Representative sections are shown (scale bar: 300 μm). (**B**) Effect of genotype and diet on steatosis in female mice. *p_TT_* = 0.050, *p_MFSD_* = 0.56, *p_FASD_* = 0.013, *p_TT x MFSD_* = 0.78, *p_TT x FASD_* = 0.015. n = 7–10/group; ordinal logistic regression, post hoc by mvt. * CC vs. TT, *p* < 0.05; # diet comparison, *p* < 0.05. (**C**) Steatosis scoring in female mice was confirmed by liver triglyceride content. n = 7–10 per diet–genotype group; *p* = 0.0012, 1-way ANOVA. CD: control diet; MFSD: methylfolate-supplemented diet; FASD: folic-acid-supplemented diet.

Hepatic steatosis was scored in Oil-Red-O-stained liver sections (Figure 3A) and analyzed by ordinal logistic regression. As in our previous study, steatosis was significantly increased in CD female mice with the 677TT genotype (Figure 3B). There was no significant effect of MFSD on steatosis in CC or TT females, and FASD did not affect steatosis in TT females. In contrast, steatosis significantly increased in FASD CC females, to the point that they were not significantly different from TT mice. To independently confirm the histology scoring, liver triglycerides were measured in female liver and found to correlate with the degree of steatosis (Figure 3C). As previously observed in our recent study [30], hepatic steatosis in male mice was unaffected by the MTHFR genotype or folate intake (Figure S2).

### 3.4. FASD Decreases MTHFR Protein Expression in 677CC Females

**Figure 4:**
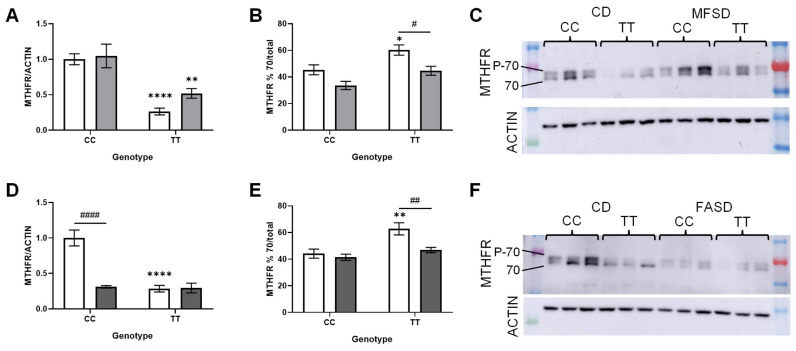
Effects of diets and genotype on MTHFR protein expression in female liver. The more active unphosphorylated 70 kDa isoform is indicated by 70, and the phosphorylated form by P-70. (**A**) MTHFR expression normalized to actin, in CD and MFSD females (*p_TT_* < 0.0001, *p_MFSD_* = 0.14, *p_interaction_* = 0.31). (**B**) % unphosphorylated 70 kDa isoform in total MTHFR in CD and MFSD females (*p_TT_* = 0.0009, *p_MFSD_* = 0.0005, *p_interaction_* = 0.59). (**C**) Representative blot for panels a and b. (**D**) MTHFR expression normalized to actin, in CD and FASD females (*p_TT_* < 0.0001, *p_FASD_* < 0.0001, *p_interaction_* < 0.0001). (**E**) % unphosphorylated 70 kDa isoform in total MTHFR in CD and FASD females (*p_TT_* =0.008, *p_FASD_* = 0.0071, *p_interaction_* = 0.049). (**F**) Representative blot for panels D and E. n = 8–9/group; 2-way ANOVA, Tukey post hoc: * CC vs. TT: * *p* < 0.05, ** *p* < 0.005, **** *p* < 0.0001. # diet: ^#^ *p* < 0.05, ^##^ *p* < 0.005, ^####^ *p* < 0.0001. Control diet (CD): white bars; methylfolate-supplemented diet (MFSD): light grey bars; folic-acid-supplemented diet (FASD): dark grey bars.

**Figure 5:**
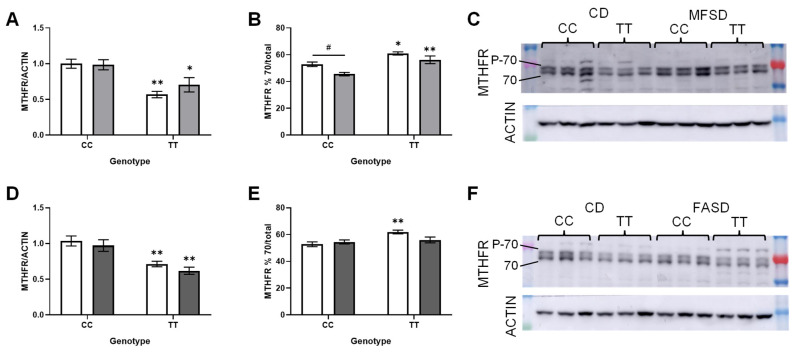
Effects of diet and genotype on MTHFR protein expression in male liver. The more active unphosphorylated 70 kDa isoform is indicated by 70, and the phosphorylated form by P-70. (**A**) MTHFR expression normalized to actin, in CD and MFSD males (*p_TT_* < 0.0001, *p_MFSD_* = 0.41, *p_interaction_* = 0.31). (**B**) % unphosphorylated 70 kDa isoform in total MTHFR in CD and MFSD males (*p_TT_* < 0.0001, *p_MFSD_* = 0.0037, *p_interaction_* = 0.51). (**C**) Representative blot for panels a and b. (**D**) MTHFR expression normalized to actin, in CD and FASD males (*p_TT_* < 0.0001, *p_MFSD_* = 0.21, *p_interaction_* = 0.81). (**E**) % unphosphorylated 70 kDa isoform in total MTHFR in CD and FASD males (*p_TT_* =0.0040, *p_FASD_* = 0.23, *p_interaction_* = 0.032). (**F**) Representative blot for panels D and E. n = 8–9/group; 2-way ANOVA, Tukey post hoc: * CC vs. TT: * *p* < 0.05, ** *p* < 0.005. # diet: ^#^ *p* < 0.05. Control diet (CD): white bars; methylfolate- supplemented diet (MFSD): light grey bars; folic-acid-supplemented diet (FASD): dark grey bars.

We have previously shown that elevated folic acid intake decreases MTHFR protein levels [22]. Therefore, the MTHFR protein expression was assessed by Western blot in both sexes (Figure 4 and Figure 5). MTHFR protein levels were significantly lower in TT mice as compared to CC (Figure 4A,D and Figure 5A,D), particularly in females (Figure 4A,D). Neither MFSD nor FASD restored the TT protein expression to wild-type levels, although the MTHFR expression in MFSD TT females was increased compared to CD TT females (*p* = 0.0070, *t*-test). MFSD had no effect on the MTHFR expression in CC males or females. In contrast, the MTHFR expression in FASD CC females was decreased to the same level as that in the TT females.

The 70 kDa subunit of MTHFR can be found in phosphorylated and unphosphorylated forms; the unphosphorylated form may be more active and/or more sensitive to SAM-regulation [43,44,45,46,47]. The proportion of the unphosphorylated 70 kDa subunit was increased in CD TT mice as compared to CD CC in both sexes, although to a greater extent in females (Figure 4B,E and Figure 5B,E). Supplementation with methylTHF or folic acid decreased this proportion in TT mice to the level seen in CC mice. This shift may be a compensatory response to the reduced methylTHF production in TT mice, a response that is corrected by increased folate intake.

### 3.5. Effect of 677TT and Supplementation on Hepatic Methylation Metabolites

**Figure 6:**
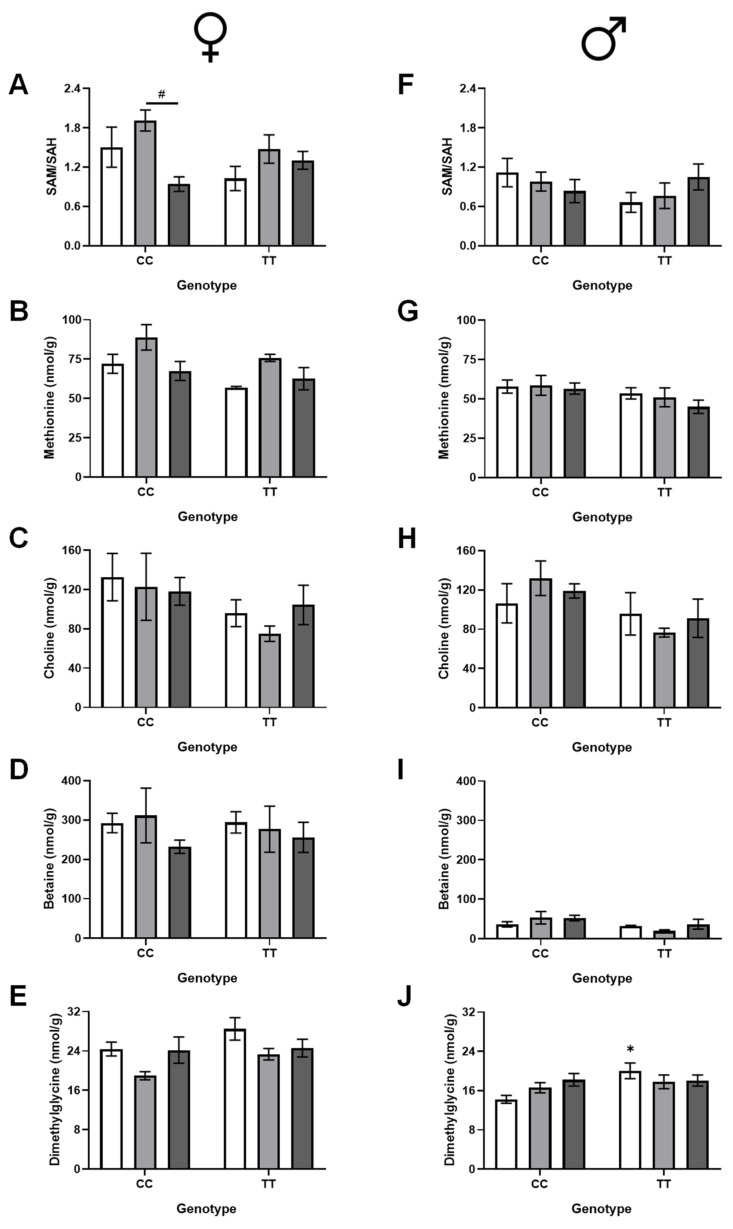
Effects of diet and genotype on methylation metabolites in liver. In females: (**A**) S-adenosylmethionine/S-adenosylhomocysteine (SAM/SAH, a measure of methylation potential; *p_TT_* = 0.27, *p_diet_* = 0.022, *p_interaction_* = 0.086), (**B**) methionine (product of Hcy remethylation, used to make SAM; *p_TT_* = 0.029, *p_diet_* = 0.0062, *p_interaction_* = 0.66), (**C**) choline (methyl donor for Hcy remethylation via betaine; *p_TT_* = 0.063, *p_diet_* = 0.74, *p_interaction_* = 0.71), (**D**) betaine (methyl donor derived from choline; *p_TT_* = 0.93, *p_diet_* =0.43, *p_interaction_* = 0.79), (**E**) dimethylglycine (product of Hcy remethylation by betaine; *p_TT_* = 0.054, *p_diet_* =0.021, *p_interaction_* = 0.48). In males: (**F**) SAM/SAH (*p_TT_* = 0.32, *p_diet_* = 0.92, *p_interaction_* = 0.19), (**G**) methionine (*p_TT_* = 0.054, *p_diet_* =0.55, *p_interaction_* = 0.75), (**H**) choline (*p_TT_* = 0.032, *p_diet_* =0.97, *p_interaction_* = 0.43), (**I**) betaine (*p_TT_* = 0.029, *p_diet_* =0.51, *p_interaction_* = 0.34), and (**J**) dimethylglycine (*p_TT_* = 0.031, *p_diet_* =0.67, *p_interaction_* = 0.056). n = 5–6/group; 2-way ANOVA, Tukey post hoc: * CC vs. TT, *p* < 0.05; ^#^ MFSD vs. FASD, *p* < 0.05. Control diet (CD): white bars; methylfolate-supplemented diet (MFSD): light grey bars; folic-acid-supplemented diet (FASD): dark grey bars.

Key metabolites in the methylation cycle (SAM, SAH, and methionine) and in folate-independent homocysteine remethylation (choline, betaine, and dimethylglycine) were measured in the male and female liver (Figure 6). In general, the concentrations of these metabolites were similar in males and females, with the notable exception of betaine, which was much lower in males than females (Figure 6D,I).

The methylation potential, as measured by the SAM/SAH ratio, was affected by folate intake in female mice, but not in males (Figure 6A,F). The marked decrease in the SAM/SAH ratio in FASD CC females is consistent with the decreased MTHFR protein expression in these mice.

Methionine concentrations were lower in TT mice of both sexes (borderline for males), but were only affected by diet in females (Figure 6B,G). In pairwise comparisons using two-way ANOVA for genotype and diet, MFSD significantly increased methionine in females (*p_TT_* = 0.018, *p_MFSD_* = 0.0038, *p_interaction_* = 0.85), whereas FASD had no significant effect (*p_TT_* = 0.10, *p_FASD_* = 0.92, *p_interaction_* = 0.39).

In females, choline concentrations tended to be lower in TT mice but were not significantly affected by folate intake (Figure 6C). The concentrations of the choline derivative betaine were unchanged by diet or genotype (Figure 6D), while those of the betaine derivative dimethylglycine tended to be higher in female TT mice and were significantly affected by diet (Figure 6E). Similar to methionine, in pairwise comparisons for females by two-way ANOVA for genotype and diet, MFSD had a significant effect on dimethylglycine (*p_TT_* = 0.011, *p_MFSD_* = 0.0023, *p_interaction_* = 0.93), while FASD did not (*p_TT_* = 0.29, *p_FASD_* = 0.34, *p_interaction_* = 0.39). Dimethylglycine is produced when betaine is used for homocysteine remethylation by betaine homocysteine methyltransferase (BHMT). Increased dimethylglycine in TT mice may reflect the increased use of the BHMT pathway to compensate for decreased methylTHF for Hcy remethylation; this increase is not required in the methylTHF-supplemented females. In males, the concentrations of all three of these metabolites were significantly altered by the TT genotype but were not influenced by folate intake.

### 3.6. Effect of 677TT and Supplementation on Expression of Key Folate Genes in Female Liver

The expression of the *Mthfr* gene in the female liver was assessed by qRT-PCR to determine if the observed decrease in MTHFR protein in the FASD CC females was a pre- or post-transcriptional effect. There were no significant changes in *Mthfr* mRNA levels due to either the TT genotype or the supplemented diets (Figure S3A), indicating that *Mthfr* transcription was unaffected by FASD. The expression of *Dhfr* and *Mtr* was similarly unaffected by genotype and diet (Figure S3B,C).

## 4. Discussion

The thermolability of the A222V MTHFR protein produced by the human *MTHFR*^677C>T^ variant has been shown to be rescued by FAD and methylTHF using the recombinant human protein [8]. Similarly, health risks associated with the 677TT genotype may be reduced by increased dietary or supplemental folates [9,10,11,12]. In this study, we demonstrated that the mouse TT protein in liver was stabilized by the MTHFR cofactor FAD, by its methylTHF product, and also by THF, which is a folate form that is neither a substrate nor a product of MTHFR. Stabilization by cofactors suggests that the 677C>T mouse model can show interactions between the dietary/supplemental intake of folates and riboflavin, in contrast to the *Mthfr*^+/−^ mouse. In this study, we compared the effects of supplementation with equivalent amounts of folic acid or methylTHF in male and female 677CC and TT mice.

CC and TT mice of both sexes were placed on a control diet, or diets supplemented with either methylTHF or folic acid. Both MFSD and FASD successfully raised circulating methylTHF; however, plasma methylTHF in TT mice remained lower than CC mice on the same diet, particularly in males. A similar effect in humans was reported in a meta-analysis of folic acid intervention studies: serum folate in 677TT individuals increased following folic acid supplementation, but remained lower than that in 677CT and CC individuals [10]. We also observed that plasma methylTHF was higher in the MFSD mice than the FASD mice. This has also been observed in women in randomized control trials of folic acid vs. methylTHF [14,16].

Although both MFSD and FASD increased plasma methylTHF, neither diet lowered plasma tHcy in TT mice. In contrast, in our previous study of folate deficiency in the 677C>T model, we showed that tHcy was significantly lower in TT mice fed the 2 mg folic acid/kg CD diet than those fed a diet containing 0.3 mg folic acid/kg diet [31]. Therefore, although plasma homocysteine is responsive to folate intake in this model, supplementation over the 2 mg folic acid/kg diet in CD did not further lower tHcy. This is similar to a meta-analysis of human studies that found that the homocysteine-lowering effect of folic acid supplements plateaued at doses of over 0.8 mg/day [48]. This plateau effect suggests that there is a metabolic bottleneck that renders folate intakes over recommended levels ineffective for tHcy lowering. However, as tHcy did not increase in the FASD CC females in this study, our findings in liver suggest that tHcy may not be a reliable indicator of perturbations of folate metabolism in the liver.

The effect of the TT genotype on hepatic methylation metabolites was relatively subtle, and the folate intake/supplement form only had significant effects in females. In the subgroup analysis, only MFSD significantly increased methionine and decreased dimethylglycine. This finding suggests that methylTHF could be immediately used for methionine production by methionine synthase (MTR), while alleviating the production of dimethylglycine by BHMT. In contrast, folic acid requires multiple conversion steps, including the reduction to methylTHF by MTHFR, before it can be used. This suggests that, in females, supplemental methylTHF may be more easily integrated into intracellular folate pools than folic acid. However, these metabolic changes were small and not sufficient to significantly affect steatosis in MFSD TT females.

A striking difference that we observed between FASD and MFSD was the specific decrease in MTHFR protein in the liver of FASD CC females. This effect was not due to a change in the gene transcription of *Mthfr*, suggesting that there are post-transcriptional or post-translational mechanisms regulating MTHFR protein levels. Possible mechanisms include interference from folate- or sex-hormone-responsive miRNAs [49,50,51], or a variety of post-translational modifications that may promote enzyme degradation [52]. The investigation of this mechanism and its sex-specificity is an interesting area for future study.

We first reported this “pseudo-MTHFR deficiency” in the liver of BALB/c background males fed a 20 mg folic acid/kg diet (2× the FASD in this study), indicating that the excess folic acid intake mimicked the effects of a genetic deficiency of MTHFR [22]. We subsequently observed this effect in the livers of pregnant FASD-fed BALB/c females [21], and in livers of FASD-fed lactating C57BL/6 females and their pups, but not in the pup brain [24]. Since we had not previously performed a direct comparison of the effects of FASD on MTHFR protein in the adult C57BL/6 liver, we were surprised to find that this effect was sex-specific. This specific decrease in MTHFR protein in the CC FASD female liver was consistent with their decreased SAM/SAH ratios. A pseudo-MTHFR deficiency has also been observed in the testes of BALB/c males fed a 40 mg folic acid/kg diet (4× FASD), but not 20 mg folic acid/kg [23], which suggests that the effect may also be dose- and tissue-dependent. One of the outstanding issues in the discussion of the risks of excess folic acid intake has been the need to distinguish the effects of excess folic acid from those of high total folate status [29]. Based on our results in this study, the deleterious effects may be due to excess folic acid.

Folate supplementation over the amount in CD had no effect on hepatic steatosis in the TT females, regardless of the folate form. However, folic acid supplementation led to increased steatosis in 677CC females, which was consistent with the reduced MTHFR protein expression and SAM/SAH ratios in these mice, which would alter the methylation potential. Essentially, the decrease in MTHFR expression in the FASD CC females to the same level as that in the TT mice resulted in an equivalent risk of steatosis. It is possible that this increase in steatosis is mediated by epigenetic changes in gene methylation, such as those observed in *Mthfr^+/−^* mice and other mice fed high-folic-acid diets [22,23,25,53]. In our previous study, we established that female TT mice had underlying expression changes in lipid metabolism genes that could impair VLDL synthesis or export and predispose the mice to hepatic steatosis [30]. Similar perturbations in lipid metabolism have been observed in zebrafish embryos; a recent study reported increased lipid accumulation in both folic-acid-supplemented larvae and in *mthfr*-deficient larvae [54].

The MTHFR expression showed a small increase in MFSD TT females; therefore, it is possible that higher levels of methylTHF supplementation may further increase the MTHFR expression. The timing of the intervention may also be important; in rodents, low folate intake during gestation has been found to increase offspring adiposity [55], while folic acid supplementation reduced offspring adiposity [56]. These findings, and those of the zebrafish, suggest that the programming of lipid metabolism, and, by extension, susceptibility to steatosis and increased adiposity, may be established in utero. Therefore, pre-natal methylTHF supplementation could be beneficial in reducing the risk of steatosis in 677TT females. Alternatively, supplementation with the FAD-precursor riboflavin has been reported to lower blood pressure in 677TT individuals [57]; a combination of methylTHF and riboflavin supplementation could further stabilize the variant protein and impact steatosis.

## 5. Conclusions

MethylTHF supplementation had limited benefits for *Mthfr*^677TT^ mice for the metabolic parameters examined in this study, and did not reduce the risk of steatosis. In contrast, folic acid supplementation had negative effects on control female 677CC mice through decreased MTHFR protein and increased steatosis. Under the conditions in this study, methylTHF was the safer supplement for female mice. Further investigation is required to explore other avenues to ameliorate steatosis in TT mice and to determine whether pseudo-MTHFR deficiency and steatosis due to excess folic acid intake occurs in human populations.

## Data Availability

All relevant data are included in this article and Supplemental Materials.

## Supplementary Materials

The following supporting information can be downloaded at: https://www.mdpi.com/article/10.3390/nu17010082/s1, Figure S1: Key enzymes and metabolites in the one-carbon folate pathways; Figure S2: Steatosis in male mice is not affected by folate intake or 677TT genotype; Figure S3: Effects of diets and genotype on expression of the *Mthfr*, *Dhfr*, and *Mtr* genes in female liver; Table S1: Formulation of experimental diets; Table S2: Primers and reaction conditions for quantitative real-time PCR.
