# Supplementary material for: Folic Acid and Methyltetrahydrofolate Supplementation in the Mthfr677C>T Mouse Model with Hepatic Steatosis
Source: Nutrients. 2024 Dec 28;17(1):82. doi: 10.3390/nu17010082 (PMC11723006; doi:10.3390/nu17010082)
Supplement: Supplementary file 1 [file nutrients-17-00082-s001.zip › nutrients-3353170-supplementary.pdf]

Online Supporting Material

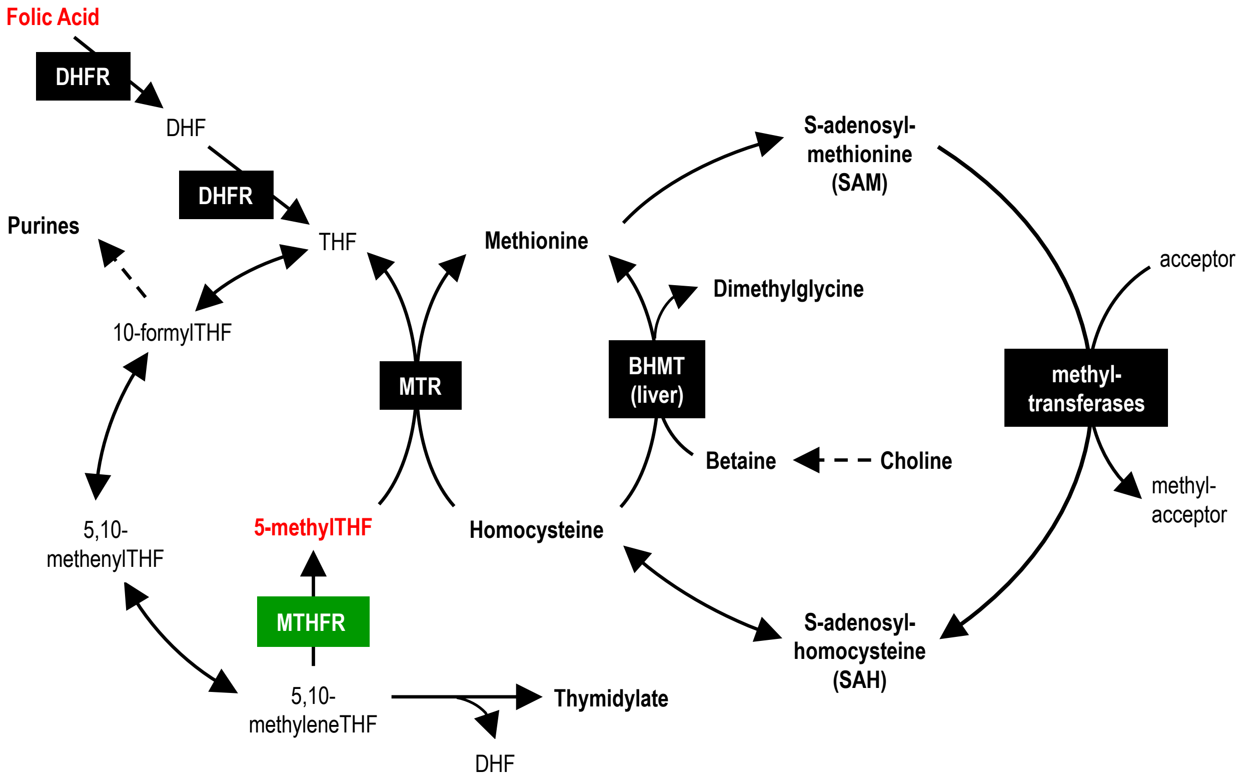

Figure S1. Key enzymes and metabolites in the one-carbon folate pathways. MTHFR is shown in green, and the 2 supplements, folic acid and methylTHF, are shown in red. In liver, BHMT provides a folate-independent pathway for homocysteine remethylation.

Online Supporting Material

Table S1: Formulation of experimental diets[1]

Diet Composition (g/kg)	CD (TD.01369) (2 mg/kg folic acid)	FASD (TD.08278) (10 mg/kg folic acid)	MFSD (TD.08278) (10.1 mg/kg Ca-methylTHF + 0.3 mg/kg folic acid)
L-alanine	3.5	3.5	3.5
L-arginine HCl	12.1	12.1	12.1
L-asparagine	6.0	6.0	6.0
L-aspartic acid	3.5	3.5	3.5
L-cystine	3.5	3.5	3.5
L-glutamic acid	40.0	40.0	40.0
Glycine	23.3	23.3	23.3
L-histidine HCl, monohydrate	4.5	4.5	4.5
L-isoleucine	8.2	8.2	8.2
L-leucine	11.1	11.1	11.1
L-lysine HCl	18.0	18.0	18.0
L-methionine	3.3	3.3	3.3
L-phenylalanine	7.5	7.5	7.5
L-proline	3.5	3.5	3.5
L-serine	3.5	3.5	3.5
L-threonine	8.2	8.2	8.2
L-tryptophan	1.8	1.8	1.8
L-tyrosine	5.0	5.0	5.0
L-valine	8.2	8.2	8.2
Sucrose	359.28[2]	349.42	359.18
Corn starch	150.0	150.0	150.0
Maltodextrin	150.0	150.0	150.0
Soybean oil	80.0	80.0	80.0
Cellulose	30.0	30.0	28.62
Mineral Mix, AIN-93M-MX	35.0	35.0	35.0
Calcium phosphate, monobasic, monohydrate	8.2	8.2	8.2
Succinylsulfathiazole	10.0	10.0	10.0
Niacin	0.03[3]	0.03[3]	0.03
Calcium pantothenate	0.016[3]	0.016[3]	0.016
Pyridoxine HCl	0.007[3]	0.007[3]	0.007
Thiamin HCl	0.006[3]	0.006[3]	0.006
Riboflavin	0.006[3]	0.006[3]	0.006
Folic acid	0.002[3]	0.010[4]	0.0003
Methyltetrahydrofolate supplement	-	-	1.385[5]
Biotin	0.0002[3]	0.0002[3]	0.0002
Vitamin B_{12} (0.1% in mannitol)	0.025[3]	0.025[3]	0.025
Vitamin E, DL-alpha tocopheryl acetate (500 IU/g)	0.15[3]	0.15[3]	0.15
Vitamin A palmitate (500,000 IU/g)	0.008[3]	0.008[3]	0.008
Vitamin D_3, cholecalciferol (500,000 IU/g)	0.002[3]	0.002[3]	0.002
Vitamin K_1, phylloquinone	0.0008[3]	0.0008[3]	0.0008
Choline bitartrate	2.5	2.5	2.5
Vitamin K, menadione sodium bisulfite	0.05	0.05	0.05
tert-Butylhydroquinone (TBHQ) antioxidant	0.02	0.02	0.02
Red food color	-	0.1	0.067
Blue food color	-	-	0.033

[1] Diet formulation is based on TD.99366 (a standard amino acid defined diet; Inotiv). Vitamin, mineral and choline content as recommended for AIN-93 (Reeves PG. *J Nutr* 1997; 127:838S-41S); amino acid content is based on Rogers QR and Harper AE. *J Nutr* 1965; 87:267-73. Methionine content is lower; however, total methionine + cysteine content exceeds the minimum in the NRC guidelines (National Research Council. Nutrient Requirements of Laboratory Animals: 4th ed. 1995).

[2] Sucrose = 349.53 g/kg added sucrose + 9.75 g/kg from Vitamin Mix AIN-93-VX

[3] Obtained from 10 g/kg Vitamin Mix AIN-93-VX

[4] Folic acid = 0.002g/kg from 10 g/kg Vitamin Mix AIN-93-VX + 0.008 g/kg added folic acid

[5] Methyltetrahydrofolate supplement (Pure Encapsulations) = 0.0101 g/kg calcium L-5-methyltetrahydrofolate + 1.375 g/kg hydroxypropyl methylcellulose

Online Supporting Material

Table S2: Primers and reaction conditions for quantitative real-time PCR (qRT-PCR)

Gene Symbol	Reference Sequence(s)	Primer Sequences	Amplicon Size (bp)	T_{ann} (°C)	[Primer] (µmol/L)
Sdha	NM_023281.1	TGTTCCGTGTGGGGAGTGTA<br>TCCAAACCATTCCCCTGTCG	102	60	0.5
Psmc4	NM_011874.2	TGGAAGACTATGTGGCCCGT<br>TCACGGACAGCCAACATTCC	88	60	0.5
Dhfr	NM_010049.3	TTGGCAAGAACGGAGACCTAC<br>ACCAGATTCTGTTTACCTTCCACTG	103	62	0.5
Mtr	NM_001081128.3	GAACACTTGGCCTACCGGAT<br>TCCAGCCACAAACCTCTTGA	102	60	0.5
Mthfr	NM_001161798.1<br>NM_010840.3	CTACCACATCGTGGACGTGA<br>GCTGGATGATCTCTCGACCC	104	60	0.5

Online Supporting Material

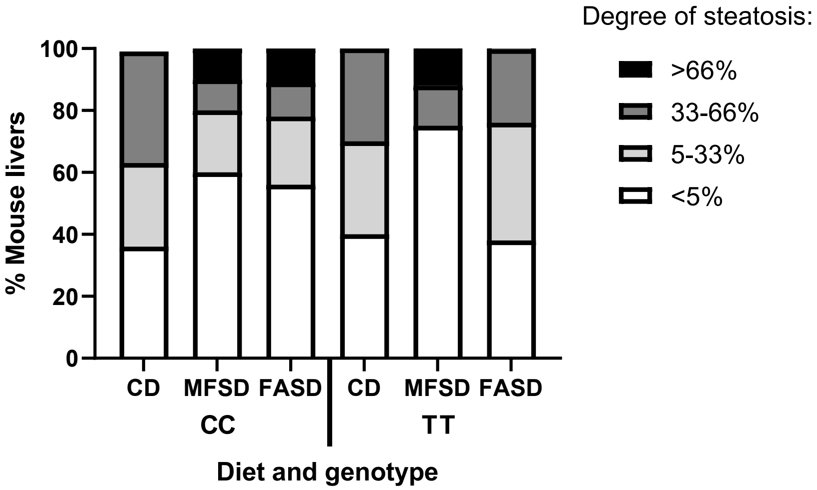

Figure S2. Steatosis in male mice is not affected by folate intake or 677TT genotype. p_{TT} = 0.89, p_{MFSD} = 0.17, p_{FASD} = 0.61. n = 8-11/group; ordinal logistic regression, post hoc by mvt. CD: control diet, MFSD: methylfolate supplemented diet, FASD: folic acid supplemented diet.

Online Supporting Material

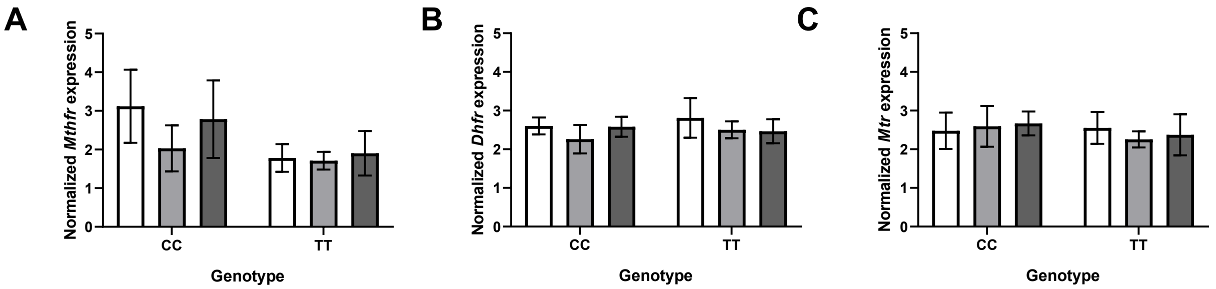

Figure S3. Effects of diets and genotype on expression of the *Mthfr*, *Dhfr*, and *Mtr* genes in female liver. (**a**) *Mthfr* (p_{TT}= 0.16, p_{diet}= 0.70, $p_{interaction}$ = 0.78), (**b**) *Dhfr* (p_{TT}= 0.68, p_{diet}= 0.61, $p_{interaction}$ = 0.83), (**c**) *Mtr* (p_{TT}= 0.60, p_{diet}= 0.97, $p_{interaction}$ = 0.87). n=6-7/group; 2-way ANOVA, Tukey post-hoc. Control diet (CD): white bars, methylfolate supplemented diet (MFSD): light grey bars, folic acid supplemented diet (FASD): dark grey bars.
